# Integrating Negative-Pressure Wound Therapy in the Therapeutic Protocol of Extensive Pediatric Burns: Current Practice and Further Treatment Decision Algorithm

**DOI:** 10.3390/medicina62050852

**Published:** 2026-04-30

**Authors:** Doina Iulia Nacea, Dan Mircea Enescu, Mihaela Pertea, Petruța Mitrache, Iulia Mihaela Gavrila, Raluca Tatar

**Affiliations:** 1 Department of Plastic Reconstructive Surgery, “Grigore Alexandrescu” Clinical Emergency Hospital for Children, “Carol Davila” University of Medicine and Pharmacy, 020021 Bucharest, Romania; iulia.nacea@umfcd.ro (D.I.N.); raluca.tatar@umfcd.ro (R.T.); 2 Department of Plastic Reconstructive Surgery and Burns, “Grigore Alexandrescu” Clinical Emergency Hospital for Children, 010621 Bucharest, Romania; 3 Department Plastic Surgery and Reconstructive, Faculty of Medicine, “Grigore T. Popa” University of Medicine and Pharmacy, 700115 Iasi, Romania; 4 Department of Plastic Surgery and Reconstructive Microsurgery, “Sf. Spiridon” Emergency County Hospital, 700111 Iasi, Romania; 5 Department of Plastic Reconstructive Surgery, “Saint John” Clinical Emergency Hospital, “Carol Davila” University of Medicine and Pharmacy, 020021 Bucharest, Romania; 6 Department of Plastic Reconstructive Surgery, “Saint John” Clinical Emergency Hospital, 042122 Bucharest, Romania

**Keywords:** pediatric burns, extensive burns, negative-pressure wound therapy, burn local treatment, electrical burns

## Abstract

*Background and Objectives*: Extensive burns are devastating injuries, especially in children, associating high risk of morbidity and mortality in the absence of immediate and appropriate treatment. Negative-pressure wound therapy (NPWT) has emerged as a versatile tool for the local treatment of burn wounds. This study aims to present our approach in using NPWT for extensive burns in children, emphasizing the indications and outcomes of these very challenging cases, and proposing an algorithm for NPWT use for extensive burn patients, even in low-resource settings. *Materials and Methods*: We retrospectively analyzed pediatric burn patients admitted between January 2020 and December 2024, selecting the cases with at least 20% TBSA burn and the application of NPWT during treatment, recording indications and parameters of use, treatment period, and results. *Results*: We identified 12 patients with a burn surface ranging from 20% to 80% TBSA, caused by high-voltage electrical current (6 cases), flame (4 cases), and scalds (2 cases). NWPT was used for 3–25% TBSA for obtaining granulation tissue in very deep burn wounds with bone and tendon exposure, for reducing edema and enhancing spontaneous re-epithelialization in intermediate circumferential burns, and for preparing the wound bed for re-grafting after local infection and graft failure. There were no complications related with the NPWT use and no fatalities. *Conclusions*: NPWT represents a reliable option for several clinical situations in local burn treatment, for temporary closure of burn areas, graft fixation, burn wound preparation, local infection control, or enhancing re-epithelialization. The proposed algorithm offers a comprehensive overview of indications of NPWT for burn local management and may guide clinical decisions, easing the identification of the best situation and moment to use the device. Our study contributes to the body of knowledge that enforces the evidence of the safe and effective use of NPWT for burn management in the pediatric population.

## 1. Introduction

Burns are devastating injuries, caused by a diversity of agents, associating high risk of morbidity and mortality in the absence of immediate and appropriate treatment. Although for some time there has been a decreasing trend in their incidence, especially in developed countries [1], they continue to represent a significant public health issue worldwide and an important burden for every healthcare system [2]. Advances in fluid resuscitation protocols and local treatment strategies have improved survival rates, together with functional and aesthetic outcomes for burn patients [3]. Nonetheless, extensive burns, where the injury encompasses 20% of total body surface area (TBSA) or more, still raise difficulties for burn teams, regarding the optimal systemic and local therapeutic approach [4].

Children are at particular risk of suffering burn injuries [5] because of insufficient or ineffective caregiver supervision and because of their natural curiosity and lack of awareness regarding dangerous situations [1]. Pediatric burns pose additional challenges to burn specialists, because children have thinner skin, an immature and fragile immune system, and low thermoregulation capacity, as well as a higher skin surface reported to the body mass, exposing them to easier dehydration [6,7,8]. Moreover, the incidence of severe burn in children proved to be more stable over time, even in the presence of very disturbing global problems, like the recent COVID-19 pandemic [9].

For the local treatment of burn injuries, there are multiple conservative and surgical options that burn teams have to integrate in a tailored approach for each patient. Initially developed in the 1990s for chronic, hard-to-heal and complex wounds of various etiologies [10] (pressure ulcers, leg ulcers, sternal and abdominal wounds, skull defects, post fasciotomy closures, lymphorrhea, bones infections, post-traumatic bone or tendon exposure, dehiscence of surgical wounds, pyoderma gangrenosum, etc. [11]), negative-pressure wound therapy (NPWT), also known as vacuum-assisted closure (VAC), has also reached a regular use in burn care since the first reported application two decades ago [12].

The main benefits of NPWT are decrease in edema, improvement of wound blood circulation, stimulation of cell proliferation and granulation of the wound bed, reduction of bacterial load, and faster engraftment [11,13,14,15,16]. These effects are obtained after applying sub-atmospheric pressure to the wound through a non-invasive, active treatment system [17]. This method does not replace surgical debridement or surgical closure. Instead, it offers bridges between different surgical stages planned for a patient in a simpler way and is associated with less morbidity related to the donor area of distant or free flaps [10].

Despite having proven benefits and multiple indications for the local treatment of various burn wounds [18], in different stages of treatment, NPWT has, so far, not become part of the standard of care procedures for burn patients. This situation can be explained by the uneven resource availability in different settings and also by the great variations registered in burn patient profiles around the world. While some centers report regular and extensive use of NPWT [19,20], others may still be looking for the best way to include this procedure in their armamentarium.

Most of the published literature on this subject consists in retrospective studies, case series, case reports, and expert opinions. Only few randomized control trials have been conducted for the use of NPWT in burn care in adult patients [21,22,23] and even fewer for children [24,25], but they are mainly focused on small or medium-sized burns. Incidentally, it would be very difficult, both from the medical and from the ethical perspective, to design randomized control trials for severe burn cases that present high mortality risk. Therefore, retrospective studies are still a valuable resource for specialized experience in clinical settings [7], especially when dealing with extensive burn patients.

Considering these, the aim of our study is to present our approach, as a national referral center for pediatric burns, in using NPWT for extensive burns in children, emphasizing the indications and outcomes of these very challenging cases. At the same time, we aim to propose an algorithm for NPWT use that could allow for consideration of this technique as a reliable therapeutic option for extensive burn patients, even in low-resource settings.

## 2. Materials and Methods

### 2.1. Study Design and Setting

This study is a retrospective descriptive cohort study conducted at a national referral center for pediatric burn care. This study analyzed pediatric patients treated with negative-pressure wound therapy (NPWT) for extensive burns between 1 January 2020 and 31 December 2024.

The primary objective of this study was to describe the real-world practice of negative-pressure wound therapy (NPWT) use in children with extensive burns in a tertiary referral center where a standardized NPWT management protocol has not been formally established. The secondary objective was to propose a structured clinical algorithm for NPWT use in extensive pediatric burns, based on our center’s experience.

The study was approved by the Institutional Ethics Committee (Approval No. 2986/20 January 2025) and was conducted in accordance with the Declaration of Helsinki. Written informed consent for treatment and data use was obtained from the legal guardians of all patients.

### 2.2. Patient Selection

All pediatric patients admitted with burn injuries during the study period were screened using International Statistical Classification of Diseases and Related Health Problems, 10th Revision, Australian Modification (ICD 10 AM) codes for burn injuries (T20.0–T28.4 and T31.20–T31.99) [26]. For each patient, the management protocol was screened to identify patients who received NPWT during hospitalization.

Inclusion criteria were:

• Hospitalization for burn injury within the study period;
• NPWT used at any stage of the management;
• Burn extent of ≥20% total body surface area (TBSA).

Exclusion criteria were:

• Absence of NPWT during the treatment course;
• NPWT used for burns <20% TBSA;
• Incomplete medical records;
• Cases outside of the study period.

**Figure 1:**
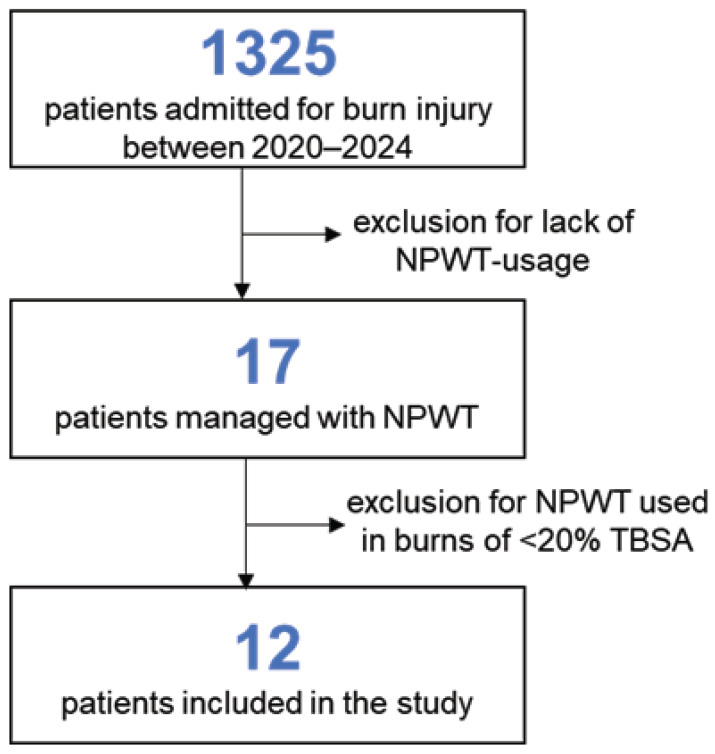
Patient flow selection for study inclusion.

The patient selection flow is depicted in Figure 1.

### 2.3. Data Collection

Clinical data were extracted retrospectively from electronic medical records and operative reports. The following variables were recorded: demographic data (age, sex), burn characteristics (etiology, TBSA, depth, anatomical distribution), intensive care unit (ICU) admission, surgical procedures performed, complications occurring during hospitalization, and total length of hospital stay.

Details collected specifically to NPWT use were timing of first application (days from admission), anatomical location and surface area treated, number of NPWT sessions, pressure settings and suction mode (continuous or intermittent), duration of therapy, local outcomes, and subsequent coverage method.

### 2.4. NPWT Usage Practice/Device Application and Settings

All patients were treated using the Vivano^®^Tec Pro NPWT system (Hartmann, Heidenheim, Germany). After surgical debridement and wound cleansing with antiseptic solutions, a silver-coated polyamide tulle was placed directly on the wound surface, as an interface layer. A polyurethane foam was then placed over the interface layer and tailored to wound dimensions. The dressing was secured using adhesive-sealing films. In anatomically challenging areas (e.g., joints, digits), hydrocolloid dressings were used as adjuncts to facilitate airtight sealing. For limb applications, a “sandwich” technique was employed, combining adhesive films and hydrocolloid barriers to ensure seal integrity and minimize air leakage.

Negative pressure parameters were selected according to wound characteristics and therapeutic goals. Continuous suction with −120 mmHg was applied for circumferential deep partial thickness burns with significant edema, or wounds treated for edema reduction and promotion of spontaneous re-epithelialization. Intermittent suction with −70/−120 mmHg intermittent mode was used for wounds with exposed bone or tendon requiring granulation tissue formation. Intermittent therapy consisted of −120 mmHg for 5 min (on phase) followed by −70 mmHg for 2 min (off phase). This cycle was repeated continuously during therapy, resulting in uninterrupted daily NPWT exposure.

### 2.5. Outcome Definitions

Successful granulation tissue formation was defined as the presence of a uniform, well vascularized granulation tissue layer adequately covering exposed bone and/or tendon, allowing definitive coverage by split thickness skin graft (STSG) or flap without the need for further debridement.

Spontaneous re-epithelialization was defined as progressive epithelial coverage of the burn wound under conservative management and NPWT, resulting in complete wound closure without surgical grafting.

Partial spontaneous re-epithelialization was defined as epithelial coverage insufficient for complete wound closure, requiring complementary STSG for definitive treatment.

A successful graft take was defined as ≥95% graft survival at first postoperative assessment following STSG, without the need for regrafting.

NPWT treatment success was defined as achievement of the intended clinical goal for which NPWT was applied (granulation tissue formation, edema reduction with re-epithelialization, or wound bed preparation after graft loss) and time to definitive wound closure, defined as the number of days from injury to final wound coverage (spontaneous re-epithelialization or surgical coverage), which was recorded retrospectively from medical files.

### 2.6. Statistical Analysis

Descriptive analysis of data was performed using Microsoft Excel 2016 data-analysis pack. Continuous variables are presented as means, medians, and ranges, while categorical variables are reported as frequencies and percentages. Due to the limited sample size and heterogeneity of burn etiology and indications, subgroup analyses were descriptive only and intended to support clinical interpretation rather than statistical inference.

For descriptive purposes, patients were stratified according to the primary clinical indication for NPWT, as follows:

1. Bridge therapy for exposed bone and/or tendon;
2. Acute edema control and prevention of burn depth progression;
3. Wound bed preparation after graft loss or local infection.

Outcomes, time to event variables, and resource utilization metrics were summarized separately for each indication group.

## 3. Results

### 3.1. Global Patient Series Description

After reviewing the hospital electronic database and the medical records of burn patients, we recorded a total number of pediatric burn admissions of 1325 cases, out of which 270 cases had at least 20% TBSA burn lesions. After screening the treatment protocol for all admitted cases, we retrieved a total of 17 patients that had NPWT in their therapeutic protocol during the study period. Since five of those cases had burn surfaces less than 20% TBSA, the total number of patients meting the inclusion criteria was 12 (Figure 1). A summary of patient data is presented in Table 1.

The majority of the patients in our series were boys, with a male-to-female ratio of 3:1. The average age was of 11 years and 3 months (median 13 years, range from 4 to 16 years old). Except for one case, all patients were admitted by transfer from other territorial hospitals due to the increased complexity of the cases, since our department is the national referral center for pediatric burns.

**Figure 2:**
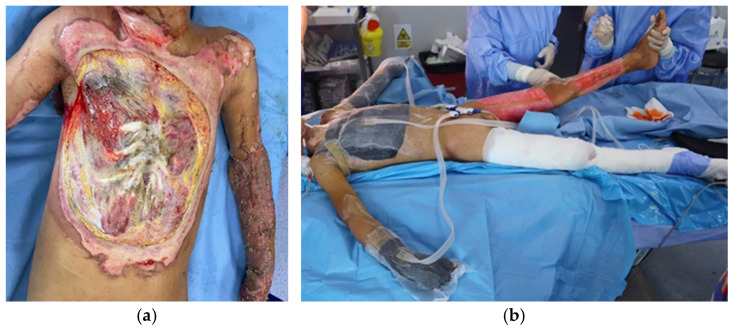

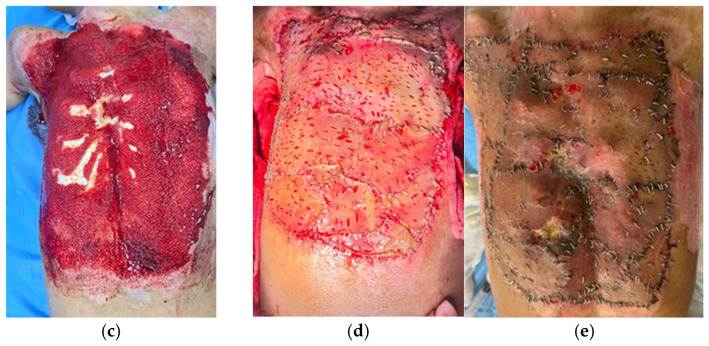
(**a**) Anterior thorax of a 10-year-old boy admitted for 50% TBSA high-voltage electrical burns (contact with power lines) with sternal, ribs, and muscular destruction; (**b**) after 2 sessions of NPWT; (**c**) after 8 sessions of NPWT, before grafting; (**d**) three days after skin grafting; (**e**) 10 days after skin grafting, with complete graft take.

**Figure 3:**
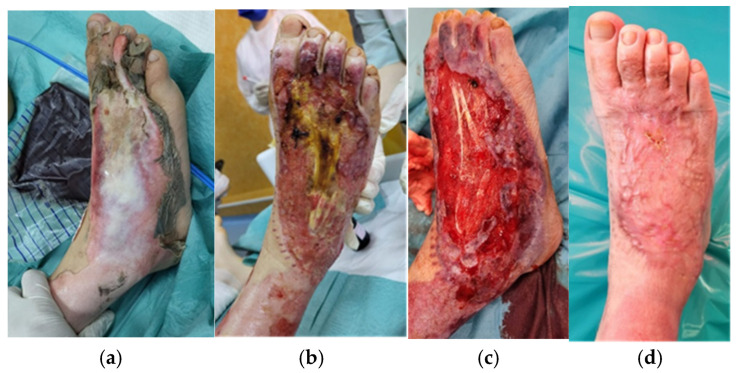
The right foot of a 12-year-old boy hospitalized with 80% TBSA high-voltage electrical burns after train climbing. (**a**) admission day; (**b**) after excision and grafting surgery, with partial graft lost and exposure of the burned extensor tendon; (**c**) after debridement, excision and two NPWT sessions, with healthy granulation tissue, before re-grafting; (**d**) 6 weeks after re-grafting, completely healed.

**Figure 4:**
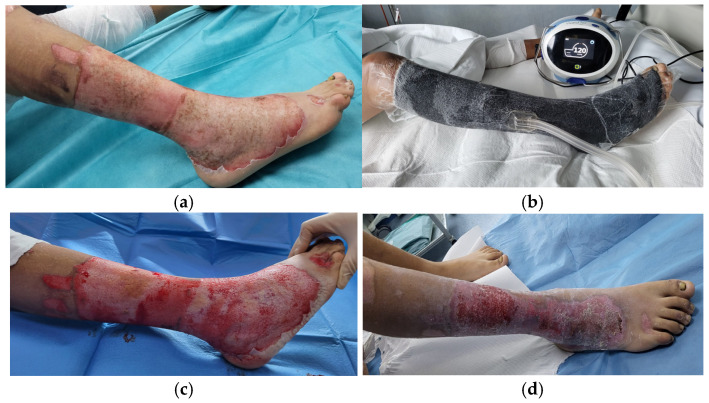
Calf and foot circular deep partial and full thickness scald burns (**a**) at admission, (**b**) while having NPWT in place; (**c**) after 5 days of NPWT (**d**) after 23 days of hospitalization with almost complete spontaneous re-epithelialization being achieved.

Regarding burn etiology, half of the patients suffered injuries through exposure to high-voltage electrical current, two of them by direct contact with the power lines (Figure 2), and the other four were train climbers that sustained trauma by falling from the top of the train (Figure 3). One-third of the cases (n = 4) were flame burns, and the rest were scalds (n = 2) (Figure 4).

Global burn depth in this series included intermediate (Figure 4), deep, and very deep wounds (the last ones having fascial, muscle, tendon and bone involvement, Figure 2 and Figure 3). Burn extent varied from 20% to 80% TBSA, with 50% of cases having suffered massive burns of more than 50% TBSA. The average TBSA was 45.3% (median 47.5%).

Considering burn extent and severity, all patients were, at first, stated on fluid resuscitation treatment, while being admitted in the ICU (except for one case, who stayed only in the burn ward). Eight cases needed escharotomies due to the presence of deep circumferential burns and risk of developing compartment syndrome.

### 3.2. NPWT Indications and Application Patterns

Since extensive burn treatment protocol has to be individualized to the particulars of every single patient, tailoring the intervention to the clinical features and also to the available resources, the NPWT application was decided based of these principles. The device and additional materials were acquired on-demand, because, for these patients, the surgical team considered that other available surgical or conservative approaches were not sufficient or effective enough to provide wound insolation, coverage, and definitive closure.

#### 3.2.1. Bridge Therapy for Exposed Bone and/or Tendon

Local assessment of the burn wounds revealed deep wounds with bone and tendon destruction from exposure to high-voltage current in 6 out of the 12 cases. This was the first indication for NPWT application, used to boost the development of granulation tissue before grafting or re-grafting. For this group of patients, the moment of device placement varied from admission to day 30 of hospitalization. For obtaining the desired results, we applied between 2 and 8 NPWT sessions per patient. An intermittent negative pressure of −120 mmHg with −70 mmHg was set. The suction dressings were changed every 5 days. For coverage, split thickness skin grafts (STSGs) were used in four of the cases and muscle flaps (one local and one free transfer) with STSGs in the other 2 cases. The grafts were subsequently covered with simple moist dressings or tie-over dressings. The average graft take was 97%.

#### 3.2.2. Acute Edema Control and Prevention of Burn Depth Progression

As a second indication, we recorded the use of NPWT for deep partial circumferential burns with significant edema, with the aim of reducing the need of escharotomies and for increasing spontaneous re-epithelialization rate. For these patients (n = 5), the negative-pressure dressings were placed between the 2nd and the 6th day from the accident. They required between one and four NPWT sessions. A continuous negative pressure of −120 mmHg was set. Each dressing was kept for 5 days. In three cases, complete spontaneous re-epithelialization was achieved under NPWT. For the other two children, we had partial spontaneous re-epithelialization, and the complete coverage was obtained with STSG. The average graft take was 99%.

#### 3.2.3. Wound Bed Preparation After Graft Loss or Local Infection

For the third clinical situation found in our patient group, we decided to include NPWT in the local treatment at a later time during the hospital stay, which was when we noticed graft take failure caused by local infectious complications. This occurred in a patient who sustained a 70% TBSA high-voltage electrical burn that had shown local wound changes three weeks post-burn on the already-excised and grafted areas on his anterior trunk. On local swab cultures, it proved to be a local infection caused by *Pseudomonas aeruginosa,* which led to partial graft loss. After thorough surgical debridement, the NPWT was applied. After two sessions of suction dressings, a significant improvement in the local appearance was obtained, with negative swabs, which allowed successful re-grafting of the resulting granulation tissue.

#### 3.2.4. Additional Details Regarding NPWT Application and Use

NPWT application and dressing changes were performed under general anesthesia or analgo-sedation, depending on burn extent, anatomical location, intended surgical maneuvers, and expected procedural discomfort. General anesthesia was preferred in more extensive or multi-site NPWT applications and procedures combined with surgical debridement or grafting. Analgo-sedation was used in isolated NPWT dressing changes without additional surgical procedures. This approach aimed to ensure patient comfort, immobility during dressing placement, and optimal seal quality. Dressings were changed every 3–5 days, and NPWT was continued until predefined clinical goals were achieved (adequate granulation tissue, spontaneous re-epithelialization, or preparation for grafting). After debridement of the lesions and cleaning with antiseptic solutions, we applied a silver-coated polyamide lattice tulle on the wound, which was covered by a polyurethane foam. We attached these first two layers by stapling them. To facilitate vacuum sealing at the limbs level, adhesive films were applied in a sandwich mode. For areas where sealing was challenging, hydrocolloid dressings have been used successfully. The body surfaces on which NPWT was applied ranged between 3 and 25%.

Data regarding NPWT indication for use, technical details, and outcome are summarized in Table 2.

### 3.3. Further Treatment and Associated Complications

For all patients included in this study, NPWT was not the only technique applied in the local treatment of the burn wounds. It was used in association with conservative dressings and excision-grafting surgeries, as part of a complex approach for extensive burn injuries affecting various and sometimes distant anatomical regions.

We performed a thorough daily clinical surveillance for NPWT-related adverse events since we started using this technique. The possible complications related with NPWT use are local bleeding under the dressing, periwound skin breakdown, local pain, local infection, and device malfunction. None of these NPWT-related adverse events were recorded in the study group. All complications observed during hospitalization were attributable to burn severity and systemic injury rather than to NPWT application. One case, a victim of with high-voltage electrical burn (direct contact with power lines), developed rhabdomyolysis syndrome, acute renal failure, and elevated cardiac enzymes upon admission. He required emergency amputations of both upper hands and forearms. Other registered complications were acute pulmonary edema (n = 2), upper gastrointestinal bleeding (n = 1), local wound infection (n = 4, isolated microorganisms: *Acinetobacter baumani*, *Aspergillus fumigatus*, and *Pseudomonas aeruginosa*), and sepsis with *Candida albicans* (n = 1), situations that prolonged their hospitalization time.

All patients benefited from an individualized functional rehabilitation program as part of their comprehensive therapeutic protocol. The early initiation of rehabilitation interventions was aimed primarily at preventing musculoskeletal complications, frequently observed in patients with extensive burns, such as scar contractures, joint ankylosis, and loss of mobility in the affected segments. The protocol comprised corrective positioning of the involved segments, intermittent use of positioning orthoses, and alternating sessions of passive and assisted active mobilization. Therapeutic positioning was maintained during rest periods, whereas controlled mobilization was performed daily, ensuring preservation of the NPWT device integrity and dressing stability. Early mobilization contributed to the stimulation of local circulation, reduction of joint stiffness, and maintenance of muscular trophicity, without compromising the wound healing process promoted by NPWT.

**Figure 5:**
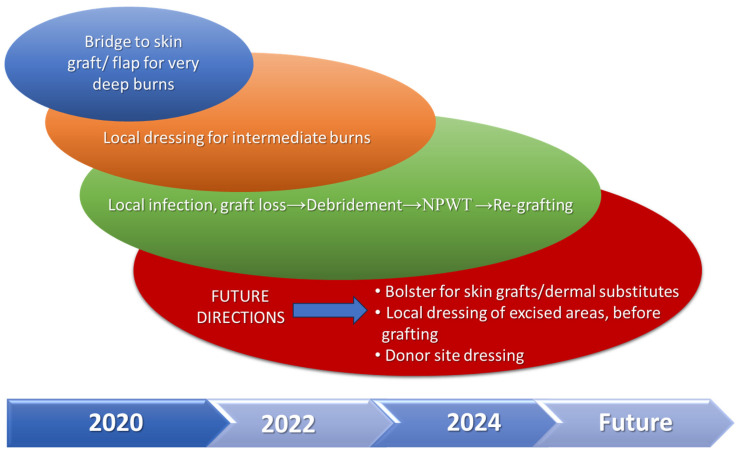
Expanding indications for NPWT use in clinical practice.

There were no fatalities in the study group. The total length of hospital stay ranged from 20 to 122 days, with a mean of 68 days. A summary of the main features for every indication, including time to definitive wound closure for the areas where NPWT was applied is presented in Table 3. Figure 5 shows a synthesis of the way we extended indications for NPWT use during the 5 years of the study and proposed further directions of use.

## 4. Discussion

### 4.1. Main Findings

When dealing with severe burn cases in children, the burn team faces, from the very first moment, a double challenge. The first priority is fluid resuscitation that aims at compensating the burn shock and SIRS and giving the patient the best chance for survival. At the same time, the local wounds are assessed and the plastic surgeons plan how to address each burned region, depending on etiology, burn depth, anatomic location, and surface extension, according to the available local resources. This approach leads to the use of multiple strategies for the same patient, adapted to the general state and local extent of the injury.

The epidemiologic data of the patients included in this research were similar to other reports in the literature, stating that pediatric burns occur more frequently in boys than in girls, that scalds are very common in infants and toddlers up to 4–5 years of age, and that older children and teenager suffer more severe burns, usually caused by flame or electricity [27,28].

Even if the total number of extensive burn patients during the study time was higher than those included in the study, we constantly have to assess and balance the available resources and treatment costs in order to give the best care to all admitted burn patients. Therefore, costly options, like NPWT, are employed only in selected cases and are available on-demand. A broader availability of the device would surely lead to an increased number of patients benefiting from this technique, based on the expanding indications mentioned above.

NPWT is a versatile and relatively easy-to-use technique, with extensive application in various surgical fields (trauma, orthopedic surgery, abdominal surgery, cardiac surgery, plastic surgery, gynecological surgery, hard-to-heal chronic wounds) [29]. Its introduction in medical practice has simplified the treatment for complex lesions and has gained a wide spectrum of indications in burn therapy as well [30]. In our department, which is a national referral center for pediatric burns and also the first plastic surgery department for children in the country, the first indication for NPWT use was as an intermediate stage between debridement and final closure, reserved only for very deep electrical burn areas or lower limb trauma cases, associated with tendon and bone exposure and extensive soft tissue loss [31]. Half of the patients included in this study had the same indication of use, as a bridge to skin graft or flap surgery for local coverage of 3rd and 4th degree burn areas, as a regional approach in the context of extensive burn injuries, mostly caused by electricity. Using NPWT in these very challenging cases offers a more conservative option that may, in the end, be covered with STSG with greater success rates and less donor site morbidity when compared with flap coverage. This approach is extremely useful, especially in the context of extensive burns, which may also involve potential donor sites for free flaps, as was the case of one of our patients (Figure 1). Moreover, high-voltage electrical burns cause significant disruptions in the skin and soft tissue vascularization, which negatively affect the possibility of planning a free transfer of muscle and cutaneous tissue. In fact, the reported cases, when microsurgery was used as a reconstructive method for very deep electrical burns, generally referred to patients with lower TBSA [32], acknowledging the difficulty of performing these surgeries in extensive burns [33], like the ones in our group.

The promotion of granulation tissue development for deep burns with associated exposure of tendons and bones is a recognized indication for NPWT [34,35]. Nonetheless, the published literature focusing on the use of NPWT reveals that the technique was employed in the treatment of burn wounds for the first time for a different indication: the acute approach, aiming at reducing edema and preventing burn injury progression, while maintaining a healing environment with reduced infection risk [12]. Subsequently, there are other studies related to this approach, confirming the initial findings [36] and demonstrating a decrease in re-epithelialization time [24]. When used during the first 24–72 h post-burn, NPWT has the potential of reversing the changes occurring within the stasis zone, and thus limits burn depth progression [24,30,37]. As the present study outlines, we also started to apply this option in the management of extensive burns, for cases scald or flame burns, with intermediate depth circumferential wounds. Most cases achieved complete re-epithelialization under NPWT, while two patients required further STSG for complete healing. Even in those deeper cases, the fact that we had several NPWT sessions finally ensured grafting after tangential excision [37]. A more traditional approach would have implied fascial excision of the burn eschar, with permanent local changes of the subcutaneous tissue and a poorer esthetic and functional result in the long term. Another advantage of this indication is the reduced need for escharotomies in circumferential burns of the extremities, because NPWT reduces edema. At the same time, especially when applied on hand burns, the NPWT dressing allows proper hand positioning, in view of subsequent rehabilitation, and decreases the number of dressing changes in an anatomic region where this procedure is more difficult [12], especially in pediatric patients [25].

In the context of extensive burns involving multiple body regions, using NPWT in the first 24–72 h as local dressing on areas of intermediate depth, especially if they are located on the extremities, allows the burn team to focus on the deep 3rd or 4th degree areas and start early excision and grafting surgeries. Subsequently, the NPWT-treated areas are reassessed, and the local treatment continued according to the local aspect, with further application of another suction dressing, classical dressings, or tangential excision and grafting, when needed.

Our third clinical scenario required the application of NPWT in a later stage of extensive burn treatment, in order to obtain healthy granulation tissue and prepare the wound bed for regrafting. There was a case of initial grafting failure due to local infection with *Pseudomonas aeruginosa* in a 15-year-old boy who sustained 70% TBSA high-voltage electrical burn as a train climber. In patients with extensive burns, the presence of *Pseudomonas aeruginosa* represents one of the most frequent and serious local infectious complications [38]. Recent studies have shown that NPWT has the capacity of reducing the risk of local infection and cleaning the wound by creating a local environment that enhances immune cell functions and diminishes bacterial charge [39,40,41]. This approach accelerates the healing of infected wounds [42], a finding confirmed by our case, that registered complete graft take after debridement and NPWT dressing.

For all of our patients, NPWT application was performed under general anesthesia or analgo-sedation. Although the procedure can also be safely performed with an awake patient [6], children show higher anxiety levels related to medical procedures, and during anesthesia or sedation, the device can be properly placed over the affected area, ensuring psychological comfort and absence of pain [31]. At the same time, the dressing changes for extensive burns is usually performed under anesthesia, and the application or change of NPWT device was part of the daily local care protocol.

Apart from those three indications, there are various studies reporting other clinical situations where NPWT was employed successfully during the burn patient course of treatment. The most common situation was the use of suction dressings above skin grafts, after surgical removal of burn eschar, to promote fast and complete graft take [21,23,43,44]. Although this method ensures better graft fixation, reducing the risk of shearing forces to displace the graft and displaying a splinting-like effect [43], it has the disadvantage of higher costs when compared with other graft dressing options [30]. Similarly, NPWT was applied on dermal substitutes, with the purpose of improving their revascularization rate and the subsequent rate of STSG take [21,30]. During the same surgical time with skin grafting, NPWT may be usen not only as bolster on STSG but also to dress the donor areas. Finally, some researchers reported the use of NPWT as a temporary coverage option for burn areas, after surgical excision of the burn eschar, in order to obtain a better wound bed before final auto-grafting. This option could allow early excision and subsequent grafting with increased chances of graft take [25,30,45].

Technical advances have made possible a modification of the initial VAC device, allowing the possibility of wound cleaning with antiseptic and antibiotic solutions through an instillation system (NPWTi). This way, the bacterial load is reduced faster, ensuring a better wound bed that will undergo further debridement and grafting [20,34]. Since this type of device was not available in our hospital, we cannot report any experience with it.

One of the main advantages of suction dressings is a lower frequency of dressing changes, when compared with traditional dressings. This way, there is less need of operating room time and staff involvement, together with a lower risk of microbial contamination, all important benefits in the context of extensive burn patients that are more fragile from the very beginning. Although our patients still had daily dressing changes for the rest of the burned areas, the time employed for this was reduced, because the regions with NPWT were assessed every 3–5 days. This approach may also decrease the psychological discomfort experienced by burn patients in relation with the moment of dressing changes [22], with this kind of distress being more intense in pediatric patients.

Depending on the indication for which NPWT is being used in burns, the number of sessions may vary from one to more than five, with the maximum applications reported in the literature (seven and eight, respectively) [46,47], being related with cases of very deep electrical burns, as we also recorded in our study group. The time for keeping the same suction dressing in place ranges between 3 and 5 days [46].

Reviewing the published literature, the body surface area which can be approached by means of NPWT in burns varies a lot, depending on the age group, indication of use, and country. In adult patients, the reported TBSA ranges from 0.5% to 90%, with lower surface in randomized controlled studies [22] and the highest values in case series [19,48]. For pediatric patients, the first report is the case of a 40% TBSA flame burn in a six-year-old boy [49], but other studies only mention 0.5 to 15% TBSA [25]. In our experience, NPWT was used on surfaces ranging from 3% to 25% TBSA, with lower values in deeper burns and higher surfaces in the acute treatment of intermediate wounds for reducing edema and preventing burn depth progression. A group of researchers reported an approach they named “Total Body Wrap”, using NPWT to cover STSG applied on burn-excised surfaces extending from 22% to 35% TBSA [50] and from 22% to 60% TBSA [51], respectively. Similarly, extra-large NPWT dressings were used in patients with 15 to 60% TBSA burns to cover both grafted areas and donor sites, extending from 17 to 44% TBSA [19]. Although interesting, this approach has several disadvantages, starting with the technical difficulties of applying the devices, the need for trained medical staff, and the costs related with the simultaneous use of 2–6 NPWT devices for the same patient [30]. In resource-limited settings or in departments with a high burden from multiple complex cases, not only restricted to burns but also other difficult plastic surgery patients with extensive skin and soft tissue defects, as it is our case, such an approach is not appropriate, since the available devices are limited, and they need to be allocated to all the patients requiring this technique.

One of the main issues that did not allow the generalized use of NPWT, especially in children, is the risk of associated complications, mostly severe bleeding and possibly death. In 2011, the FDA released a safety warning in 2011 [6], but there were adult cases with NPWT applied over large vessels and associated coagulopathy [39]. This situation has no similarities with the burn wound. Despite this, there are a lot of published studies and case reports documenting the safe and effective use of NPWT, both in adult and pediatric burn patients, with only minor incidents such as minor bleeding and hematoma, mild local infection, or device-related problems, without a relevant impact on the general outcome [7]. The present study supports these findings and also our previous experience, since we have been using NPWT safely in our department for more than a decade for burns and for complex trauma cases [31].

Together, with all the mentioned benefits of NPWT in the acute stage of burn treatment, the application of sub-atmospheric pressure has a positive impact on the quality of the burn scars achieved after complete wound coverage and healing, as assessed by scar score measurements [25], especially when used for intermediate burns that do not require further grafting [17]. Considering the fact that scar quality is also influenced by the early initiation and effective management of rehabilitation procedures, is it important to underscore the fact that NPWT allows early mobilization without jeopardizing tissue repair processes [7,43,52]. In our patient group, initiation of the rehabilitation program from the first day of treatment contributed to the preservation of joint alignment and prevention of deforming positions, particularly at the level of the extremities, where the risk of post-burn contracture is substantial.

New therapies sometimes come with a decrease in hospitalization time, which represent a justification of higher costs associated with them. For NPWT, the evidence is unclear, as some researchers report shorter LOS when compared to standard of care [19,36], while others did not register any change in this parameter [22]. For extensive burns, survival is the main goal of treatment, and we did not analyze the impact of the NPWT on LOS. We could say that the duration of hospitalization for our patients was in line with our usual experience, influenced by the burn severity and defined by surface extent, depth, and associated comorbidities.

### 4.2. Proposed Algorithm for Integrating NPWT in the Extensive Burn Management Protocol

**Figure 6:**
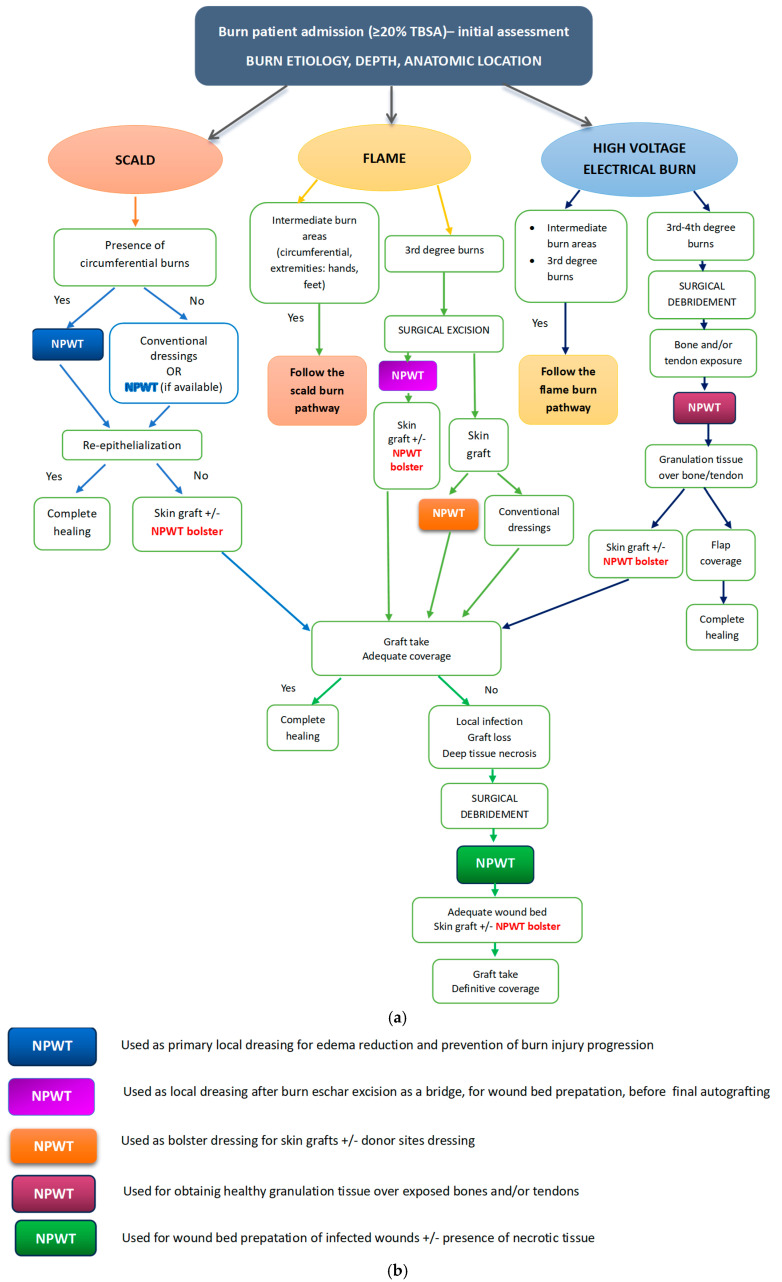
(**a**) Algorithm for integrating NPWT in the management of extensive burns. (**b**) Legend explaining the indications of NPWT use in burn treatment.

Considering all the clinical situations and details discussed above, comprising our own experience and the published literature on this topic, together with the fact that NPWT does not have standards for use in burn patients to date [53,54], we are proposing the following clinical algorithm that synthetizes all the above-mentioned therapeutic options (Figure 6a,b). The pathways are constructed following the three main causing agents of extensive burns: scalds, flame, and high-voltage electrical current.

For resource-limited settings, where NPWT has to be employed only in few, very well-chosen cases, as it was our situation during the study time, the main indication should be for obtaining healthy granulation tissue over exposed bones and/or tendons, followed by the wound bed preparation in case of skin graft loss, local infection, and necrosis. With increased financial resources and less cost-related restrictions, the indications may be subsequently expanded with the other options listed below.

### 4.3. Study Advantages, Limitations, and Further Directions

The present study introduced a comprehensive overview on the current and possible applications of NPWT in the management of severe burns in children. Although there are many articles discussing the use of this technique on STSG after burn wound excision in adult patients, only few explore other possible indications as we did. At the same time, we presented the integration of NPWT for the management of several cases of extensive high-voltage electrical burns, very challenging clinical situations that have become less frequent in other European countries [55].

The main study limitations are related to the retrospective design and the small number of patients included in the study group. Nonetheless, we have to consider the fact that extensive burns are overall less frequent than smaller surface ones [1]. Given the rarity and severity of such injuries, large prospective cohorts are difficult to obtain. Nevertheless, the present series represents real-world experience from a national referral center and provides clinically relevant insights into NPWT use in this challenging population. At the same time, the randomized controlled trials and the meta-analyses published to date either focused on small surfaces or had small-sized groups [23], since it would be very complicated from the practical and also from the ethical perspective to perform such research on extensive life-threatening burns. NPWT was not applied systematically to all extensive burn patients during the study period. Its use was based on clinical indications and pragmatic decision making by the treating team. Consequently, selection bias cannot be completely excluded. However, this real-world, indication-driven approach reflects routine clinical practice in a tertiary pediatric burn center and formed the basis for the descriptive objectives of the study and for the development of a proposed NPWT management algorithm.

Another limitation of the study is the heterogeneity of burn etiology, including flame, scald, and high-voltage electrical injuries. These mechanisms differ in depth, tissue involvement, and healing dynamics, which may influence local wound management strategies and outcomes. However, this heterogeneity reflects the real-world case mix encountered in our department and the challenges that burn teams have to face. Given the descriptive nature of the study, burn etiology was not used for comparative analysis but was documented and considered when interpreting indications and outcomes of NPWT use. The limited sample size did not allow for meaningful etiology specific subgroup comparisons.

Other limitations refer to the fact that we did not have the possibility of using NPWTi and also that we did not yet apply NPWT on skin grafts or on excised burn zones, due to local financial restrictions.

Future research should aim at confirming the safety profile of NPWT in the pediatric population. Other prospective studies could focus on the application of NPWT as temporary dressing of excised areas before grafting, granting an extensive early burn wound removal and a comparison of costs, safety, and efficacy with other types of available local dressings.

## 5. Conclusions

Successful management of extensive burns requires sustained efforts and a tailored approach for each individual patient. NPWT represents a reliable option for several clinical situations in local burn treatment, for temporary closure of burn areas, graft fixation, for burn wound preparation, for local infection control, or for enhancing re-epithelialization. Depending on resource availability, it can be safely used on extensive areas or only on restricted, very challenging and deep burn surfaces. The proposed algorithm offers a comprehensive overview of indications of NPWT for burn local management and may guide clinical decisions in specialized burn centers, easing the identification of the best situation and moment to use the device. By exploring the outcomes of extensive burns in children, our study contributes to the body of knowledge that reinforces the evidence of the safe and effective use of NPWT for burn management in the pediatric population, despite previous warnings and restrictions.

## Figures and Tables

**Table 1:** Patients’ demographic and epidemiological data.

Patient No.	Age (Years)	Gender	Burn Etiology	TBSA (%)	Maximum Depth (Burn Degree)	ICU Stay (Days)	Total LOS (Days)
1	14	F	Flame	30	3rd	1	40
2	16	M	High-voltage electrical burns—power lines	20	4th	3	122
3	16	M	High-voltage electrical burns—train	70	4th	42	99
4	4	M	Scald	50	3rd	5	57
5	4	F	Scald	25	3rd	1	22
6	15	M	High-voltage electrical burns—train	75	4th	4	90
7	9	M	Flame	45	3rd	1	64
8	5	F	Flame	45	3rd	2	53
9	15	M	High-voltage electrical burns—train	70	4th	12	96
10	10	M	High-voltage electrical burns—power lines	50	4th	6	80
11	12	M	High-voltage electrical burns—train	80	4th	10	72
12	15	M	Flame	20	3rd	0	20

**Table 2:** NPWT indications and technical details of use.

Patient No.	NPWT Indication	NPWT First Application Day (Since Admission)	NPWT Place	Number of NPWT Sessions	NPWT Settings	Results	Covering Method
1	partial tendon exposure	5	one upper limb	1	−120 mmHg continuous	Partial spontaneous re-epithelialization	STSG
2	bones and tendons exposure	23	hand + both feet	3	−70/120 mmHg intermittent	Granulation tissue	STSG
3	bones exposure	16	one lower limb	2	−120 mmHg continuous	Granulation tissue	Latissimus dorsi muscular free flap
4	deep partial burns	5	one lower limb	4	−120 mmHg continuous	Spontaneous re-epithelialization	None
5	edema + circular deep partial burns	2	one upper limb	1	−120 mmHg continuous	Partial spontaneous re-epithelialization	STSG
6	bones and tendons exposure	30	both lower limbs	2	−120 mmHg continuous	STSG take	muscular local flaps + STSG
7	edema + circular deep partial burns	6	one upper limb	1	−120 mmHg continuous	Spontaneous re-epithelialization	None
8	edema + circular deep partial burns	3	both upper limbs	2	−120 mmHg continuous	Partial spontaneous re-epithelialization	STSG
9	extensive pseudomonas skin infection	25	circular trunk	2	−120 mmHg continuous	healing the infection	STSG
10	bones exposure	1	anterior trunk, both upper limbs	8	−70/120 mmHg intermittent	Granulation tissue	STSG
11	bones and tendons exposure	40	one lower limb	2	−70/120 mmHg intermittent	Granulation tissue	STSG
12	edema and circular deep partial burns	2	one upper limb	2	−120 mmHg continuous	Spontaneous re-epithelialization	None

**Table 3:** Summary of NPWT application details and timeframe..

NPWT Indication Group	n	TBSA % (Median [Range])	NPWT First Day (Median [Range])	NPWT Sessions (Median [Range])	Time to Definitive Wound Closure
Bridge therapy (exposed tendon/bone)	6	60.0 [20–80]	19.5 [1–40]	2.0 [1–8]	53 [36–72]
Acute edema/progression prevention	5	45.0 [20–50]	3.0 [2–6]	2.0 [1–4]	20 [14–30]
Infected wounds/graft loss	1	70.0 [70–70]	25.0 [25–25]	2.0 [2–2]	58

## Data Availability

The original contributions presented in this study are included in the article; further inquiries can be directed to the corresponding authors. The data can be obtained from the corresponding authors upon reasonable request due to privacy concerns.

## Abbreviations

The following abbreviations are used in this manuscript:

NPWT	Negative-pressure wound therapy
VAC	Vacuum-assisted closure
NPWTi	Negative-pressure wound therapy with instillation
TBSA	Total body surface area
STSG	Split thickness skin grafts
ICU	Intensive care unit
LOS	Length of hospital stay

## References

[1] Frear C.C., Griffin B., Cuttle L., McPhail S.M., Kimble R. (2019). Study of negative pressure wound therapy as an adjunct treatment for acute burns in children (SONATA in C): Protocol for a randomised controlled trial. Trials.

[2] Badoiu S.C., Enescu D.M., Tatar R., Miricescu D., Stanescu-Spinu I.I., Greabu M., Coricovac A.M., Badoiu S.E., Jinga V. (2024). Adipokines—A Cohort Prospective Study in Children with Severe Burns. Int. J. Mol. Sci..

[3] Żwierełło W., Piorun K., Skórka-Majewicz M., Maruszewska A., Antoniewski J., Gutowska I. (2023). Burns: Classification, Pathophysiology, and Treatment: A Review. Int. J. Mol. Sci..

[4] McCann C., Watson A., Barnes D. (2022). Major burns: Part 1. Epidemiology, pathophysiology and initial management. BJA Educ..

[5] World Health Organization Burns. https://www.who.int/news-room/fact-sheets/detail/burns.

[6] Santosa K.B., Keller M., Olsen M.A., Keane A.M., Sears E.D., Snyder-Warwick A.K. (2019). Negative-Pressure Wound Therapy in Infants and Children: A Population-Based Study. J. Surg. Res..

[7] Pedrazzi N.E., Naiken S., La Scala G. (2021). Negative Pressure Wound Therapy in Pediatric Burn Patients: A Systematic Review. Adv. Wound Care.

[8] Pelizzo G., Calcaterra V., Canonica C.P.M., Magenes V.C., Marinaro M., Durante E., Cordaro E., Zuccotti G. (2025). Burns in Early Childhood: Age-Specific Causes, Risks, Management, and Implications—A Narrative Review. Children.

[9] Tatar R., Enescu D.M., Nacea D.I., Nițescu G.V., Lescaie A., Pertea M., Mitrache P., Diaconu L.S. (2025). The Effects of the COVID-19 Pandemic on Trends and Types of Pediatric Burn Injuries: Lessons from a National Burn Center and the Role of Strategic Resource Allocation. Life.

[10] Orgill D.P., Bayer L.R. (2013). Negative pressure wound therapy: Past, present and future. Int. Wound J..

[11] Lima R.V.K.S., Coltro P.S., Júnior F.J.A. (2017). Negative pressure therapy for the treatment of complex wounds. Rev. Col. Bras. Cir..

[12] Kamolz L.P., Andel H., Haslik W., Winter W., Meissl G., Frey M. (2004). Use of subatmospheric pressure therapy to prevent burn wound progression in human: First experiences. Burns.

[13] Mouës C.M., Heule F., Hovius S.E. (2011). A review of topical negative pressure therapy in wound healing: Sufficient evidence?. Am. J. Surg..

[14] Normandin S., Safran T., Winocour S., Chu C.K., Vorstenbosch J., Murphy A.M., Davison P.G. (2021). Negative Pressure Wound Therapy: Mechanism of Action and Clinical Applications. Semin. Plast. Surg..

[15] Chen L., Zhang S., Da J., Wu W., Ma F., Tang C., Li G., Zhong D., Liao B. (2021). A systematic review and meta-analysis of efficacy and safety of negative pressure wound therapy in the treatment of diabetic foot ulcer. Ann. Palliat. Med..

[16] Shi J., Gao Y., Tian J., Li J., Xu J., Mei F., Li Z. (2023). Negative pressure wound therapy for treating pressure ulcers. Cochrane Database Syst. Rev..

[17] Seth I., Gibson D., Lim B., Cevik J., Bulloch G., Xie Y., Marcaccini G., Rozen W.M., Cuomo R. (2024). Advancements, applications, and safety of negative pressure wound therapy: A comprehensive review of its impact on wound outcomes. Plast. Aesthet. Res..

[18] Nuhiji E. (2024). Trends and Innovation in Negative Pressure Wound Therapy: A Review of Burn Wound Management. Adv. Wound Care.

[19] Fischer S., Wall J., Pomahac B., Riviello R., Halvorson E.G. (2016). Extra-large negative pressure wound therapy dressings for burns—Initial experience with technique, fluid management, and outcomes. Burns.

[20] Donoso-Samper A., Camacho-Obando D., Garzón S., Gómez-Ortega V. (2024). Enhanced Negative Pressure Wound Therapy Shortens Hospital Stay for Major Burn Patients: Case Series. Plast. Reconstr. Surg. Glob. Open.

[21] Lin D.Z., Kao Y.C., Chen C., Wang H.J., Chiu W.K. (2021). Negative pressure wound therapy for burn patients: A meta-analysis and systematic review. Int. Wound J..

[22] Tapking C., Endlein J., Warszawski J., Kotsougiani-Fischer D., Gazyakan E., Hundeshagen G., Hirche C., Trofimenko D., Burkard T., Kneser U. (2024). Negative pressure wound therapy in burns: A prospective, randomized-controlled trial. Burns.

[23] Lee S.C., Bayan L., Sato A., Vankayalapati D.K., Antoniou V., Shami M.Z., Sulaiman H.O., Yap N., Nakanishi H., Than C.A. (2025). Benefits of negative pressure wound therapy in skin grafts: A systematic review and meta-analysis of randomised controlled trials. J. Plast. Reconstr. Aesthet. Surg..

[24] Frear C.C., Cuttle L., McPhail S.M., Chatfield M.D., Kimble R.M., Griffin B.R. (2020). Randomized clinical trial of negative pressure wound therapy as an adjunctive treatment for small-area thermal burns in children. Br. J. Surg..

[25] Lou J., Zhu X., Xiang Z., Fan Y., Song J., Huang N., Li J., Jin G., Cui S. (2024). The efficacy and safety of negative pressure wound therapy in paediatric burns: A systematic review and meta-analysis of randomized controlled trials. BMC Pediatr..

[26] World Health Organization (2022). The International Statistical Classification of Diseases and Related Health Problems, Twelfth Revision, Australian Modification (ICD-10-AM).

[27] Morrow S.E., Smith D.L., Cairns B.A., Howell P.D., Nakayama D.K., Peterson H.D. (1996). Etiology and outcome of pediatric burns. J. Pediatr. Surg..

[28] García-Díaz A., Gacto-Sánchez P., Durán-Romero A.J., Carrasco-García S., Ruiz-Moya A., Molina-Morales J., Sánchez-Tatay M.-V., Gómez-Cía T., Pereyra-Rodríguez J.-J. (2022). Pediatric major burns: A monocentric retrospective review of etiology and outcomes (2008–2020). Eur. J. Plast. Surg..

[29] Miller-Mikolajczyk C., Beach K., Silverman R., Cooper M. (2024). The Evolution of Commercial Negative Pressure Wound Therapy Systems over the Past Three Decades. Adv. Wound Care.

[30] Kantak N.A., Mistry R., Varon D.E., Halvorson E.G. (2017). Negative Pressure Wound Therapy for Burns. Clin. Plast. Surg..

[31] Enescu D.M., Stoicescu S., Tomiţă M., Nacea I., Ioniţă D., Tatar R. (2020). Management of lower extremity traumatic injuries with negative pressure wound therapy: Experience of a pediatric plastic surgery department. Injury.

[32] Thomson C.J., Miles D.A., Beveridge J., Chang P.S. (2004). Treatment of electrical burns by single debridement followed by free-flap coverage: How important is timing?. Can. J. Plast. Surg..

[33] Fan C., Azam F., Hinson C., Sink M., Jamison D., Awaida C., Fisher M., Odobescu A. (2025). Free Flap Reconstruction in Burns: A Systematic Review of Current Practices and Evidence. Microsurgery.

[34] Blome-Eberwein S., Lozano D., Amani H. (2018). Utility of negative pressure wound therapy with instillation in a burn center. Burns Open.

[35] Cretu A., Grosu-Bularda A., Bordeanu-Diaconescu E.M., Hodea F.V., Ratoiu V.A., Dumitru C.S., Andrei M.C., Neagu T.P., Lascar I., Hariga C.S. (2025). Strategies for Optimizing Acute Burn Wound Therapy: A Comprehensive Review. Medicina.

[36] Rosadi Seswandhana M., Anzhari S., Dachlan I., Widodo Wirohadidjojo Y., Aryandono T. (2020). A case series of negative pressure wound therapy as a promising treatment in patients with burn injury. Int. J. Surg. Case Rep..

[37] Lumsden E., Kimble R., McMillan C., Storey K., Ware R.S., Griffin B. (2023). The feasibility of negative pressure wound therapy versus standard dressings in paediatric hand and foot burns protocol: A pilot, single-centre, randomised control trial. Pilot Feasibility Stud..

[38] Roy S., Mukherjee P., Kundu S., Majumder D., Raychaudhuri V., Choudhury L. (2024). Microbial infections in burn patients. Acute Crit. Care.

[39] de Jesus L.E., Martins A.B., Oliveira P.B., Gomes F., Leve T., Dekermacher S. (2018). Negative pressure wound therapy in pediatric surgery: How and when to use. J. Pediatr. Surg..

[40] Frazee R., Manning A., Abernathy S., Isbell C., Isbell T., Kurek S., Regner J., Smith R., Papaconstantinou H. (2018). Open vs. Closed Negative Pressure Wound Therapy for Contaminated and Dirty Surgical Wounds: A Prospective Randomized Comparison. J. Am. Coll. Surg..

[41] Ibrahim Z.M., Waked I.S., Ibrahim O. (2019). Negative pressure wound therapy versus microcurrent electrical stimulation in wound healing in burns. J. Wound Care.

[42] Burhan A., Khusein N.B.A., Sebayang S.M. (2022). Effectiveness of negative pressure wound therapy on chronic wound healing: A systematic review and meta-analysis. Belitung Nurs. J..

[43] Kamolz L.P., Lumenta D.B., Parvizi D., Wiedner M., Justich I., Keck M., Pfurtscheller K., Schintler M. (2014). Skin graft fixation in severe burns: Use of topical negative pressure. Ann. Burns Fire Disasters.

[44] Hoeller M., Schintler M.V., Pfurtscheller K., Kamolz L.P., Tripolt N., Trop M. (2014). A retrospective analysis of securing autologous split-thickness skin grafts with negative pressure wound therapy in paediatric burn patients. Burns.

[45] Eyvaz K., Kement M., Balin S., Acar H., Kündeş F., Karaoz A., Civil O., Eser M., Kaptanoglu L., Vural S. (2018). Clinical evaluation of nega-tive-pressure wound therapy in the management of electrical burns. Ulus. Travma Acil Cerrahi Derg..

[46] Kement M., Başkıran A. (2018). Efficacy of negative pressure wound therapy in the management of acute burns. Ulus. Travma Acil Cerrahi Derg..

[47] Sung K.Y., Lee S.J., Joo H.S. (2020). Treatment Strategy for an Electrical Burn Patient with Multiple Fourth-Degree Wounds. J. Wound Manag. Res..

[48] Teng S.C. (2016). Use of negative pressure wound therapy in burn patients. Int. Wound J..

[49] Schintler M., Marschitz I., Trop M. (2005). The use of topical negative pressure in a paediatric patient with extensive burns. Burns.

[50] Low O.W., Chong S.J., Tan B.K. (2013). The enhanced total body wrap--the new frontier in dressing care for burns. Burns.

[51] Chong S.J., Liang W.H., Tan B.K. (2010). Use of multiple VAC devices in the management of extensive burns: The total body wrap concept. Burns.

[52] Liu C., Xie H., Wei P., Gong T., Wu G., Xu Z., Chen S. (2023). Clinical study of early rehabilitation training combined with negative pressure wound therapy for the treatment of deep partial-thickness hand burns. Front. Surg..

[53] Gómez-Ortega V., Vergara-Rodriguez M.J., Mendoza B., García T. (2021). Effect of Negative Pressure Wound Therapy in Electrical Burns. Plast. Reconstr. Surg. Glob. Open.

[54] Radzikowska-Büchner E., Łopuszyńska I., Flieger W., Tobiasz M., Maciejewski R., Flieger J. (2023). An Overview of Recent De-velopments in the Management of Burn Injuries. Int. J. Mol. Sci..

[55] Korkiamäki A., Kinnunen E., Lindford A., Vuola J. (2024). Electrical burns in train climbers treated in the Helsinki Burn Centre during the last 30 years. Scand. J. Trauma Resusc. Emerg. Med..

